# Genetic Encoding of Arylazopyrazole Phenylalanine for Optical Control of Translation

**DOI:** 10.1021/acsomega.3c03512

**Published:** 2023-07-14

**Authors:** Chasity P. Janosko, Olivia Shade, Taylor M. Courtney, Trevor J. Horst, Melinda Liu, Sagar D. Khare, Alexander Deiters

**Affiliations:** † Department of Chemistry, University of Pittsburgh, Pittsburgh, Pennsylvania 15260, United States; ‡ Department of Chemistry and Chemical Biology, Rutgers University, Piscataway, New Jersey 08854, United States

## Abstract

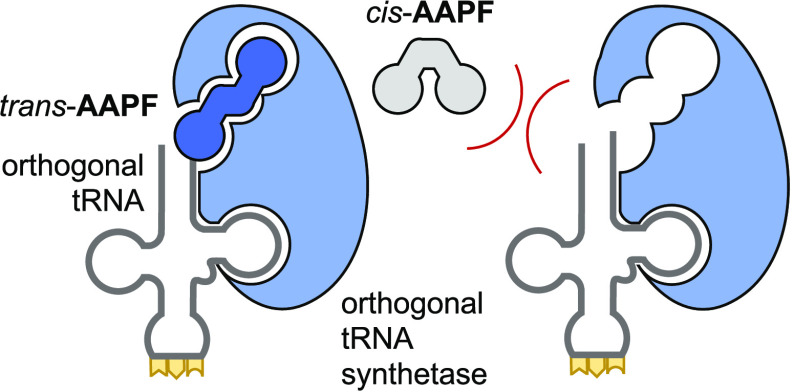

An arylazopyrazole was explored for its use as an enhanced photoswitchable amino acid in genetic code expansion. This new unnatural amino acid was successfully incorporated into proteins in both bacterial and mammalian cells. While photocontrol of translation required pulsed irradiations, complete selectivity for the *trans*-configuration by the pyrrolysyl tRNA synthetase was observed, demonstrating expression of a gene of interest selectively controlled via light exposure.

## Introduction

Optogenetics, or the genetic encoding of light-controlled proteins, allows for non-invasive investigation of biological processes with precise spatiotemporal control.^1^ Light-responsive proteins, such as phytochrome and cytochrome photoreceptors, have been used to investigate signaling pathways,^2,3^ transcription,^4,5^ and protein localization.^6−8^ While light-responsive protein domains can enable precise spatiotemporal control of the specified system, a protein fusion is always needed. Conversely, photocaged proteins, or proteins bearing a photolabile moiety that blocks a specified function before cleavage and activation with light,^9^ require only a point mutation for the introduction of an unnatural amino acid (UAA) while still maintaining precise optical control. Photocaged proteins are useful for investigating certain biological systems, but the irreversible nature of caging group removal prevents extended control of the system. Instead, reversible control of biological function is a powerful tool as it enables persistent and precise spatiotemporal control.^10^ Photoswitchable molecules use light to induce isomerization and alter protein function, and they have become a valuable alternative to photocaged systems when prolonged protein control is needed.

Genetic code expansion is one of several optogenetic approaches to placing protein function under light control.^11−14^ The genetic incorporation of a UAA is enabled by an orthogonal aminoacyl-tRNA synthetase and tRNA that recognize and suppress an amber stop codon to incorporate the UAA.^15^ Further, the *Methanosarcina mazei*, *Methanosarcina barkeri*, or *Methanomethylophilus alvus* pyrrolysyl-tRNA synthetase/tRNA_CUA_ (PylRS/PylT) pairs are favored as their tRNAs are natural amber suppressors and the pairs are orthogonal in both mammalian and *Escherichia coli* cells.^15−18^ The PylRS/PylT pair has been engineered to encode over 100 UAAs, providing a range of new chemical structures and new functionalities to proteins.^19^ With precise site specificity, genetic code expansion has found numerous applications in protein modification.^20,21^

Azobenzene-derived photoswitchable UAAs, the first being azobenzene phenylalanine (**AzoF**), have been genetically encoded and used to control DNA binding^22^ and the function of fluorescent proteins.^23^ Additionally, two fluorinated analogs of **AzoF** were shown to reversibly control the function of a luminescent protein,^24^ and photoswitchable Cys-reactive amino acids have been used to control protein function through alteration of protein conformation.^25,26^ Although current tools show the ability to photoswitch in a cellular environment and, in some cases, provide reversible control of protein function, the photostationary states (PSS) of these chromophores are not ideal. For example, **AzoF** irradiated with 365 nm light leads to a PSS of 78% *cis*, and irradiation with 450 nm light yields a PSS of 76% *trans*. Additionally, the thermal stability of *cis-***AzoF** is limited to ∼13 h.^27^ The incomplete isomerization at each wavelength and the stability of the *cis*-isomer allow ample space for improvement,^22^ as an ideal photoswitch would undergo quantitative isomerization to the specified configuration. Fluorinated analogs of **AzoF** showed enhanced *trans*-to-*cis* PSS but remained inefficient in returning to the *trans*-configuration upon irradiation with different wavelengths.^24^

In addition to the azobenzene core utilized in **AzoF**, other azo-containing heterocycles have been developed to improve photochemical properties of the azo-functionality. Small chromophores containing aryl-azo groups linked to imidazole,^28^ indazole,^29^ triazole, pyrazole, pyrrole, and tetrazole^30^ have been developed to further improve the PSS of this class of photoswitches. Namely, an arylazopyrazole derivative presented near-complete photoswitching properties, with a PSS of 98% *cis* and 98% *trans* when irradiated with 355 and 532 nm light, respectively.^30^ Despite the improved photoswitching capabilities of azo-heterocyclic chromophores, UAAs bearing these functional groups have yet to be employed in the photocontrol of proteins or peptides.

In designing UAAs for photoswitchable control of protein function, the size of the photoswitch also needs to be considered. In contrast to other photoswitchable motifs,^31^ azobenzenes and azo-heterocycles are relatively small, which facilitates recognition by a tRNA synthetase.^32^ We hypothesized that an arylazopyrazole-modified phenylalanine (**AAPF**) would show improved photophysical properties as compared to its azobenzene predecessors while maintaining the ability to be genetically encoded.

## Results and Discussion

### Synthesis of an Arylazopyrazole-Modified Phenylalanine

**Figure 1:**
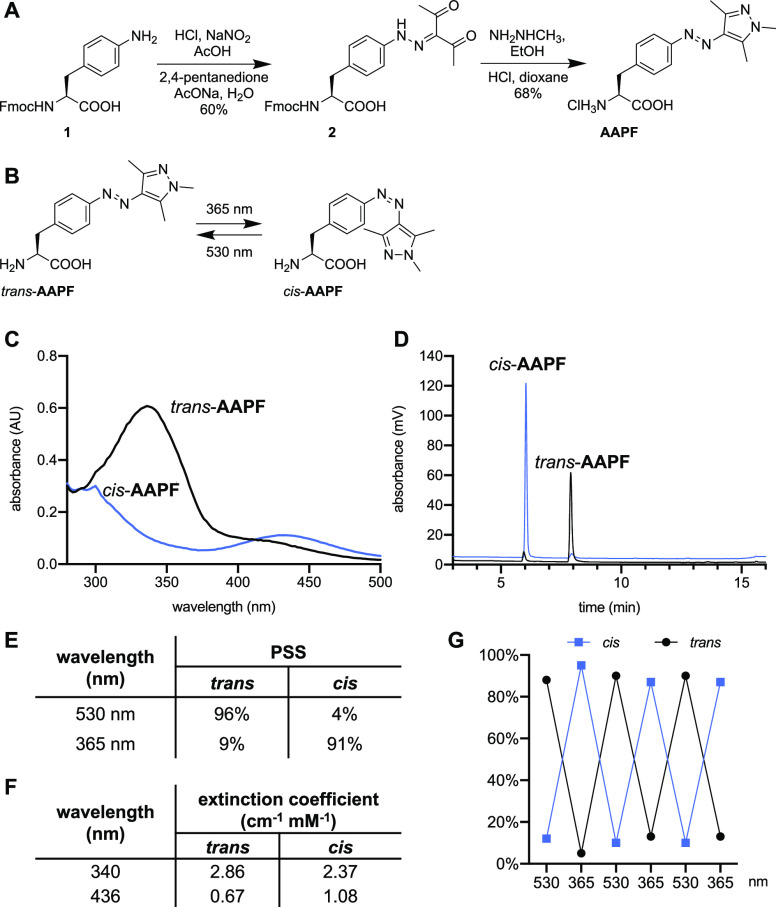
Synthesis and photochemical analysis of **AAPF**. (A) Synthesis of **AAPF**. (B) Irradiation of **AAPF** with 365 and 530 nm light converts the *trans-*isomer to the *cis-*isomer and back, respectively. (C) Absorbance spectra and (D) HPLC chromatograms (absorbance at 280 nm) of both isomers (250 μM in PBS, 2.5% DMSO) show near-complete photoswitching. (E, F) Photostationary states and extinction coefficients of **AAPF** after 10 min of irradiation at either 530 or 365 nm. (G) Repeat photoswitching of **AAPF** as measured by HPLC.

The photoswitchable **AAPF** was synthesized in two steps (Figure 1A) from the commercially available Fmoc-4-aminophenylalanine (**1**). First, the *para*-amine was converted to the corresponding diazonium salt, followed by immediate enolate addition to form hydrazone **2** in 60% yield.^33,34^ Methyl hydrazine was then utilized in both the formation of the pyrazole moiety and cleavage of the Fmoc protecting group, yielding the final product **AAPF** in 68% yield. To improve solubility for biological assays, **AAPF** was then converted to the corresponding HCl salt.

### Determination of Photostationary States

Prior to using **AAPF** in cell-based assays, we sought to confirm its ability to act as an efficient photoswitch. Based on the photoswitching properties of the corresponding 3′,5′-dimethylated arylazopyrazole,^30^ we hypothesized that irradiation with 365 nm light would provide the *cis-*isomer of **AAPF**, whereas irradiation with 530 nm light would provide the *trans-*isomer (Figure 1B). Solutions of **AAPF** (250 μM) were prepared in phosphate-buffered saline (PBS) and irradiated with either 365 nm (UV transilluminator, 10 min) or 530 nm (LED, 10 min) light. The samples were then analyzed via absorbance scan and HPLC.

The absorbance spectrum of *trans*-**AAPF** shows the two absorbance maxima characteristic of arylazo compounds,^31^ with the highest extinction coefficient near 340 nm corresponding to the symmetry-allowed π–π* transition and a smaller peak maximum near 430 nm corresponding to the symmetry-forbidden n−π* transition (Figure 1C). Upon irradiation with 365 nm light, the absorbance spectrum of **AAPF** shows a decrease in the π–π* absorption and an increase in the n−π* absorption, indicating isomerization from the *trans-* to the *cis*-isomer (Figure 1C). To quantify each photostationary state, samples were analyzed via HPLC. Two distinct peaks were observed at 6.0 and 7.9 min, corresponding to the *cis-* and *trans-*isomers, respectively. Integration of each peak area revealed near-complete photoswitching, with 96% of *trans*-**AAPF** after irradiation with 530 nm light and 91% of *cis-***AAPF** after irradiation with 365 nm light (Figure 1D,E). The molar extinction coefficient of **AAPF** was calculated by measuring the absorbance of 1 mM **AAPF** (irradiated with either 530 or 365 nm for the *trans* and *cis* states, respectively) at both of the local absorbance maxima, 436 and 340 nm (Figure 1F). Further, the reversibility of **AAPF** was validated with sequential irradiations at 530 and 365 nm and yielded consistent isomerization between the *trans-* and *cis-*isomers, respectively, as quantified by HPLC (Figure 1G). Efficient and reversible photoswitching of **AAPF** is observed by the distinct changes in the absorbance spectra and HPLC chromatograms, making **AAPF** the most efficient photoswitchable UAA to date.^22,24^

### Incorporation of **AAPF** into Protein in *E. coli*

**Figure 2:**
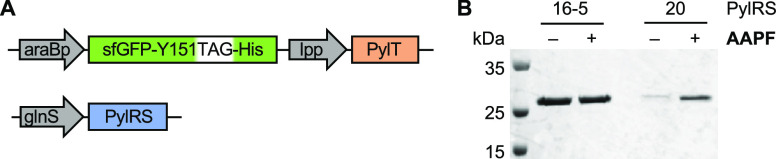
Incorporation of **AAPF** into protein in *E. coli*. (A) Two-plasmid system utilized for genetic encoding of **AAPF** in bacterial cells. (B) Expression and purification of sfGFP in the presence and absence of **AAPF** using PylRS-16-5 and PylRS-20 (AzoFRS2) analyzed via SDS-PAGE and visualized using Coomassie stain.

After successful demonstration of photoswitching, the incorporation of **AAPF** into proteins in bacterial cells was explored. To encode **AAPF**, a two-plasmid system was utilized (Figure 2A). One plasmid encodes both the gene of interest, in this case, sfGFP with an amber stop codon (TAG) at position Y151, and the pyrrolysyl tRNA (PylT). The second plasmid encodes a mutant of the *M. barkeri* pyrrolysyl synthetase (PylRS). To select a synthetase for optimal **AAPF** incorporation, a small panel of 13 PylRS mutants was screened (Table S1). Expression of the fluorescent protein sfGFP was determined for each individual culture via fluorescence intensity, which was then normalized to the absorbance at 600 nm to account for any culture-to-culture variation in the number of cells collected (Figure S1). From this synthetase screen, two potential hits were identified due to the high level of **AAPF** incorporated relative to background gene expression: PylRS-16-5 and PylRS-20.

These two hits were further analyzed by performing larger scale expressions of sfGFP-Y151**AAPF** with either PylRS-16-5 or PylRS-20. His-tagged sfGFP-Y151**AAPF** was isolated, and expression levels were analyzed by SDS-PAGE (Figure 2B). PylRS-16-5 showed high background incorporation in the absence of **AAPF**, which was confirmed by mass spectrometry. In contrast, high levels of sfGFP-Y151**AAPF** were observed with PylRS-20 and only low background incorporation of endogenous phenylalanine, as confirmed by mass spectrometry (Figure S2). PylRS-20 has previously been named AzoFRS2 for its ability to encode azobenzene analogs,^24^ and thus, it is not surprising that it was also able to accept **AAPF** as a substrate.

### Incorporation of **AAPF** into Protein in Mammalian Cells

**Figure 3:**
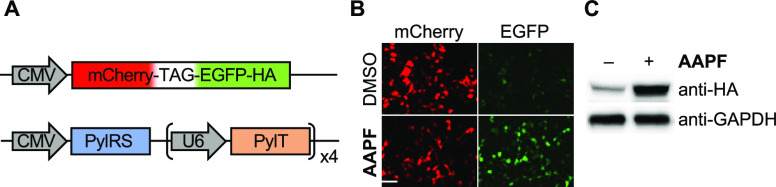
Incorporation of **AAPF** in HEK293T cells. (A) Schematic of the two-plasmid system utilized for incorporation of **AAPF** into mammalian cells. (B) HEK293T cells were analyzed for green fluorescence from EGFP expression. The scale bar is equal to 50 μm. (C) Western blot analysis of cells transfected with pmCherry-TAG-EGFP-HA in the presence or absence of **AAPF**.

We also tested incorporation of **AAPF** in mammalian cells. HEK293T cells were transfected with a two-plasmid system similar to that used in the bacterial experiments above.^15^ The first plasmid encodes an mCherry-EGFP fusion protein, designed such that the TAG stop codon is located between the two fluorescent proteins, yielding pmCherry-TAG-EGFP-HA. With this placement of the stop codon, mCherry is constitutively expressed and serves as an internal control for transfection, whereas EGFP is only expressed if **AAPF** incorporation is successful. The second plasmid encodes both the PylRS, in this case, the mammalian codon-optimized AzoFRS2, and four copies of the corresponding PylT (Figure 3A).

Fluorescence microscopy was used to validate the incorporation of **AAPF** into pmCherry-TAG-EGFP-HA expressed in mammalian cells (Figure 3B). HEK293T cells were transfected with the necessary plasmids in the presence or absence of **AAPF** (250 μM). Following a 24 h incubation, the cells were washed with live cell imaging solution and imaged for EGFP (ex. 470/40, em. 525/50 nm) and mCherry (ex. 550/25, em. 605/70 nm) fluorescence using a Zeiss fluorescence microscope. Imaging revealed EGFP fluorescence in the presence of **AAPF**, showing efficient incorporation of the UAA and expression of the full-length protein (Figure 3B).

To further validate the live cell imaging result, mammalian cells expressing mCherry-**AAPF**-EGFP-HA were lysed and analyzed via western blot (Figure 3C). With the placement of a C-terminal HA-tag on pmCherry-**AAPF**-EGFP, the anti-HA western blot confirms the expression of the full-length protein. Expression of the reporter was observed in the presence of **AAPF**, confirming successful incorporation of **AAPF** in mammalian cells using AzoFRS2. Some full-length protein was detected even in the absence of **AAPF**, which correlates well with the small amount of background observed in bacterial expressions (Figure 2B). Western blot and fluorescence microscopy both demonstrate clear incorporation of **AAPF** over background through detection of the full-length mCherry-**AAPF**-EGFP construct.

### Optical Control of Translation through Photoswitching of **AAPF**

Control of translation has been previously demonstrated using chemically modified tRNA and mRNA. Caged tRNAs have been used in the light activation of translation in cell-free systems and in mammalian cells;^35,36^ however, chemically acylated tRNAs have limited stability (<4 h) and hydrolyze readily.^36^ Photocaged mRNAs have been generated through enzymatic placement of photocaging groups in the 5′-UTR^37^ or the 5′-cap,^38^ which allowed for efficient optical control of translation in mammalian cells. Furthermore, photoswitchable chromophores integrated into the 5′-cap enabled reversible light activation of translation in cells^39^ and zebrafish embryos.^40^ While these approaches utilize innovative designs, they are limited by the short half-life of synthetic mRNA, which is estimated to range from only a few minutes to ∼9 h in mammalian cells and 4 to 5 h in zebrafish.^41−43^ We hypothesized that optical control of UAA recognition would address long-term stability concerns due to the intracellular production of mRNA and tRNA through transcription, thus providing a novel means for light-controlled protein expression. The underlying concept is the strong preference of an engineered amino-acyl synthetase to recognize only one configuration of a photoswitchable UAA as a substrate.

**Figure 4:**
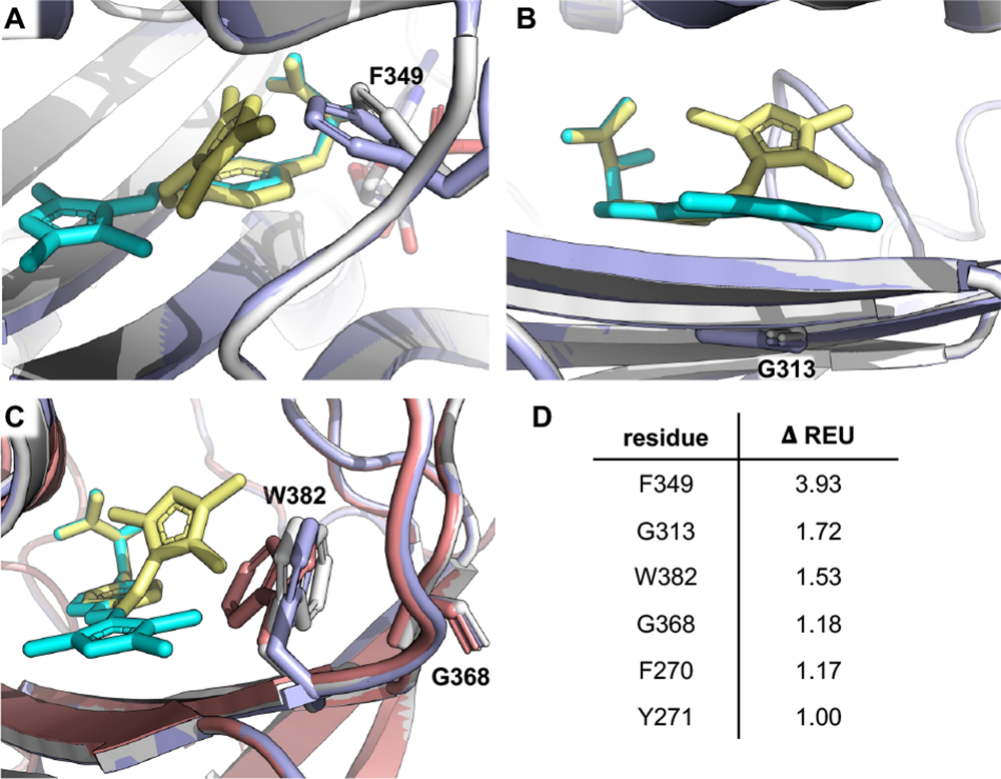
Computational analysis of *cis*- vs *trans*-**AAFP** in AzoFRS2. Effects of *cis*-**AAPF** (yellow) and *trans*-**AAPF** (cyan) on AzoFRS2 residues (A) F349, (B) G313, and (C) W382 and G368. (D) Differences in Rosetta energies for AzoFRS2 active site residues when docked with either *cis*- or *trans*-**AAPF**.

To investigate the feasibility of each **AAPF** isomer to be a substrate for AzoFRS2 (PDB: 4ZIB),^44^ computational modeling was performed using PyRosetta. First, AzoFRS2 binding site models were generated with adenosine triphosphate (ATP), three magnesium ions, a coordinating water molecule, and either isomer of **AAPF**. The total energies and computed pocket energies were then used to evaluate the feasibility of the **AAPF** isomer binding to the AzoFRS2 active site.^45^ To generate catalytically active models, a near-attack geometry was constrained between the UAA and the ATP. Per-residue energy decomposition revealed several interactions that are more stable when AzoFRS2 is docked with *trans-***AAPF** than with *cis-***AAPF**. When **AAPF** was in the *cis* configuration, the pyrazole moiety clashed with F349 of AzoFRS2, thus forcing rotation and increasing its energy by 3.93 Rosetta Energy Units (REU) (Figure 4A). Residue G313 has a favorable interaction with *trans*-**AAPF**, which was lost in the *cis* configuration, thus causing a difference of 1.72 REU (Figure 4B). Both isomers of **AAPF** lead to a rotameric change in W382 when compared to the apo model, but *cis-***AAPF** disrupted it by 1.53 REU more than the *trans-***AAPF**. This disruption further impacted G368 by an additional 1.18 REU for *cis-***AAPF** compared to *trans-***AAPF** (Figure 4C). Other residues that contributed to a more stable AzoFRS2 active site with *trans-***AAPF** rather than with *cis-***AAPF** include F270 and Y271 (Figure 4D). Overall, the energies observed upon binding of *trans-***AAPF** were 11 REU lower than the energies corresponding to *cis-***AAPF** binding, with 10 REU of the energy differential occurring within the binding pocket, indicating that *trans-***AAPF** should be strongly favored by AzoFRS2 (Table S2). With the computational evidence suggesting that AzoFRS2 favors *trans*-**AAPF** but does not entirely preclude *cis*-**AAPF** binding, we hypothesized that photocontrol of **AAPF** could be utilized to gain selective control of suppressor tRNA acylation and therefore control protein yield using irradiation.

**Figure 5:**
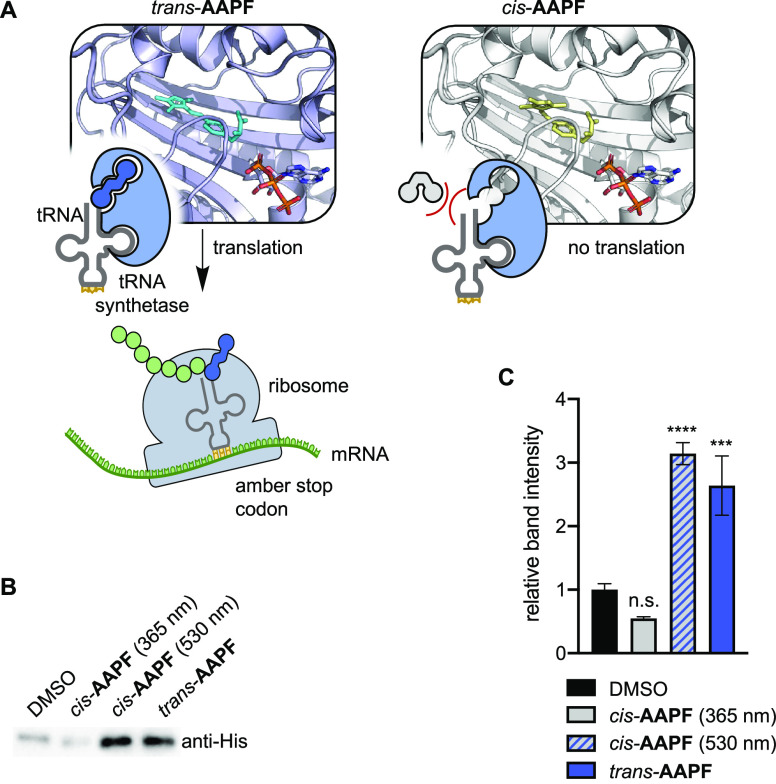
Photocontrol of translation using **AAPF**. (A) Schematic representation of selective recognition of *trans-***AAPF** by AzoFRS2 with corresponding computationally determined PylRS structures. (B) Western blot analysis of sfGFP-Y151**AAPF** expressed in *E. coli* cells treated with either *cis*-**AAPF** (irradiated with 365 nm every hour), *cis*-**AAPF** immediately irradiated with 530 nm, or *trans*-**AAPF**. (C) Quantification of western blot data from biological triplicates, where error bars represent standard deviation. Statistical analysis done via one-way ANOVA with multiple comparisons to the DMSO control, where n.s. = *p* = 0.1419, ***p ≤ 0.0001, and ****p ≤ 0.001.

Recognition of *trans*- versus *cis-***AAPF** by AzoFRS2 was then tested in bacterial cells (Figure 5A). The same two-plasmid expression system used to test **AAPF** incorporation in bacterial cells above was used to compare the incorporation of *cis*- vs *trans*-**AAPF** by AzoFRS2 via western blot analysis. *E. coli* cells transformed with pBAD-sfGFP-Y151TAG were treated with either *cis*- or *trans-***AAPF** (1 mM, pre-irradiated with either 365 or 530 nm light, respectively), and then protein expression was induced by arabinose. After an overnight incubation, each culture was analyzed by anti-His western blot to visualize expression of sfGFP-Y151**AAPF**. Interestingly, complete background incorporation of *cis*-**AAPF** was observed in this experiment (Figure S4). We hypothesized that this observed background may be due to reversion to the *trans-*isomer over the long incubation period.

To maintain the specified isomer throughout the course of the expression, a subset of the *cis-***AAPF**-treated cultures were removed from the incubator and irradiated with the 365 nm light for 5 min every hour for 6 h after induction. To demonstrate control of translation, a subset of *cis-***AAPF**-treated cultures were irradiated with 530 nm light (5 min) immediately after induction. After a total induction time of 6 h, each culture was analyzed by anti-His western blot to visualize expression of sfGFP-Y151**AAPF** (Figure 5B,C and Figure S5). While treatment with *cis-***AAPF** alone does show background translation, short intervals of irradiation with 365 nm light throughout the total time of expression completely eliminate any observed background. Further, when treated with *trans-***AAPF**, a strong mRNA translation response is observed, suggesting selective recognition of *trans-***AAPF** by AzoFRS2.

### Limited Stability of *cis*-**AAPF**

**Figure 6:**
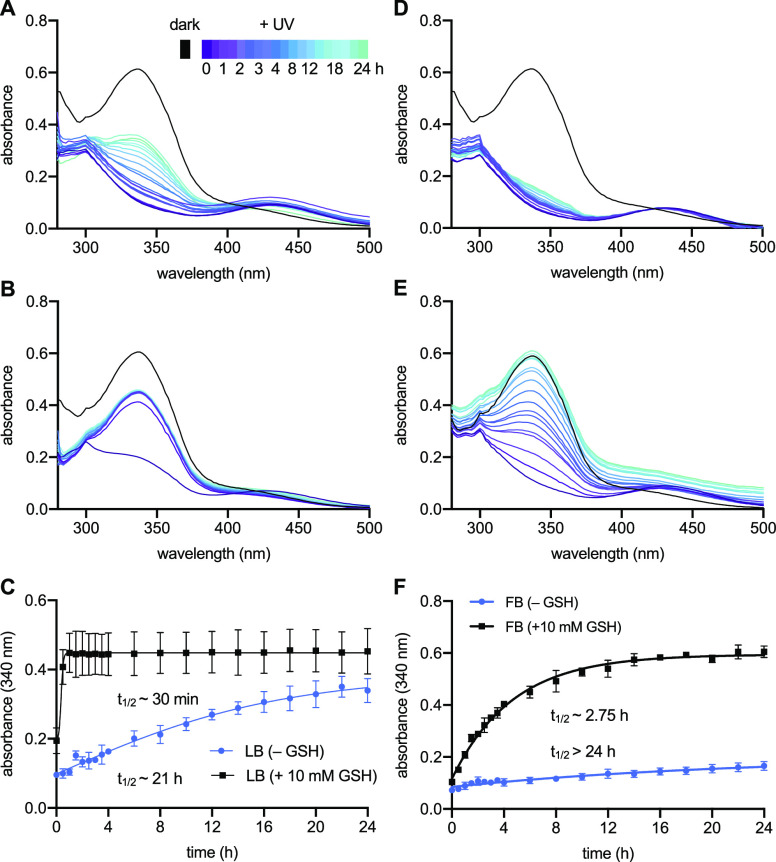
Thermal stability of *cis-***AAPF**. Absorbance spectra of *cis-***AAPF** in (A) LB broth, (B) LB broth supplemented with 10 mM GSH, (D) FluoroBrite media, or (E) FluoroBrite media supplemented with 10 mM reduced glutathione, measured in triplicate over 24 h. Change in absorbance at 340 nm (*trans*-**AAPF** λ_max_) was plotted over time for (C) LB +/– GSH and (F) FB +/– GSH, and respective *t*_1/2_ values were determined for each condition.

While the observed selectivity for *trans-***AAPF** with regularly pulsed irradiations validated our computational predictions, we further investigated the initially observed background incorporation observed in *cis-***AAPF**-treated cells during longer incubation periods. We found that the thermal stability of *cis*-**AAPF** is affected by the solvent, as decreased thermal stability was observed in acetonitrile, showing a *t*_1/2_ of 2.5 h, but stability was improved in PBS to a *t*_1/2_ of 36 h (Figure S3). The stability of *cis-***AAPF** was then monitored in LB broth and FluoroBrite media (Figure 6A,D, respectively) to more closely mimic protein expression conditions. FluoroBrite media was used as an alternative to typical DMEM to prevent phenol red interference with the absorbance spectra. For each *cis*-**AAPF** stability test, a solution of **AAPF** (250 μM) was prepared in the specified media and then irradiated with 365 nm light for 10 min to reach the *cis* PSS. Each sample was then analyzed by an absorbance scan every 30 min for the first 4 h and then every 2 h after irradiation for a total of 24 h. Over the full 24 h, a considerable thermal isomerization back to the *trans-*isomer was observed, as the intensity at 340 nm continued to increase. By plotting the absorbance at 340 nm over time, the half-lives of 21 and >24 h were determined for *cis*-**AAPF** in LB broth and FluoroBrite media, respectively (Figure 6C,F). These extended half-lives suggested that the media alone is not contributing to the observed instability of the *cis-*isomer.

For further investigation into *cis-***AAPF** stability, its sensitivity to thiol reduction, and subsequent isomerization back to *trans*-**AAPF**, was analyzed. Azobenzenes are known to undergo reduction by thiols,^46,47^ so we tested *cis*-**AAPF** stability in the presence of glutathione (GSH), which persists in cellular environments between 0.5 and 10 mM.^46^ To mimic both bacterial and mammalian expression conditions, LB broth and FluoroBrite media were supplemented with 10 mM reduced glutathione, and *cis*-**AAPF** stability was monitored by the absorbance spectra (Figure 6B,E, respectively). The presence of the thiol led to much faster relaxation of *cis-***AAPF** back to *trans*-**AAPF** in both LB broth and FluoroBrite media. From the collected absorbance spectra, *cis*-**AAPF** half-lives of 30 min and 2.75 h were determined in LB broth and FluoroBrite media, respectively (Figure 6C,F). Overall, the thermal isomerization of *cis*-**AAPF** in either media with glutathione is fast enough that a percentage of *trans*-**AAPF** will likely be formed during the duration of a typical protein expression. Because the *trans-*isomer is recognized by AzoFRS2 and expressions usually occur over several hours, the thermal relaxation of *cis*-**AAPF** back to *trans-***AAPF** in our initial experiment was likely the cause of the observed background translation. We cannot rule out that some *cis-***AAPF** may be accepted as a substrate by AzoFRS2, although it is unlikely, as the addition of regularly pulsed irradiations throughout the time of expression is sufficient to eliminate background UAA incorporation.

## Conclusions

A photoswitchable phenylalanine derivative, arylazopyrazole phenylalanine (**AAPF**), was synthesized, and its photochemical properties were characterized. **AAPF** reaches its *trans* photostationary state after 10 min of irradiation at 530 nm. When irradiated with 365 nm light for 10 min, the *cis* photostationary state is reached, yielding an absorbance spectrum with decreased π–π* absorbance at 340 nm and increased n−π* absorbance at 436 nm. The quantification of each photostationary state validates near-complete isomerization, with 96% *trans-***AAPF** and 91% *cis*-**AAPF** observed after respective irradiations. Further, the reversible isomerization of **AAPF** was demonstrated through sequential irradiations and characterization of the resultant isomer. To genetically encode this photoswitch, a tRNA synthetase panel screen revealed a PylRS, previously termed as AzoFRS2, that efficiently incorporated the amino acid into proteins in both bacterial and mammalian cells. Through molecular modeling, selective incorporation of *trans-***AAPF** by AzoFRS2 was rationalized and experimental tests demonstrated a distinct preference for *trans*-**AAPF** over the *cis*-isomer. This enabled optical control of translation of a gene of interest through the simple introduction of **AAPF** at an amber stop codon. While background activity was observed over time due to thermal reversion of the *cis*-isomer, simple addition of regularly pulsed irradiations throughout the course of the experiment eliminated background translation. Overall, this UAA shows promise as a reversible translational switch for photocontrol of gene expression. In future studies, we intend to investigate **AAPF** as an optical switch of protein function.

## Supporting Information Available

Synthetic protocols; NMR and MS characterizations; photochemical analysis protocols and results; biological protocols including synthetase screening, **AAPF** incorporation in bacterial and mammalian cells, live cell imaging, and western blots; computational protocols; thermal stability of *cis*-**AAPF** (PDF)
