# Supplementary material for: Genetic Encoding of Arylazopyrazole Phenylalanine for Optical Control of Translation
Source: ACS Omega. 2023 Jul 14;8(29):26590–6. doi: 10.1021/acsomega.3c03512 (PMC10373180; doi:10.1021/acsomega.3c03512)
Supplement: Supplementary file 1 — ao3c03512_si_001.pdf [file ao3c03512_si_001.pdf]

Supporting Information

Genetic Encoding of Arylazopyrazole Phenylalanine for Optical Control of Translation

Chasity P. Janosko,[1#] Olivia Shade,[1#] Taylor M. Courtney,[1#] Trevor J. Horst,[1] Melinda Liu,[2] Sagar D. Khare,[2] and Alexander Deiters[1*]

[1] *Department of Chemistry, University of Pittsburgh, Pittsburgh, PA 15260*
[2]*Department of Chemistry and Chemical Biology, Rutgers University, Piscataway, NJ 08854*

[#] *These authors contributed equally.*

Synthesis Protocols

General. All chemicals obtained from commercial sources ChemScene, Fisher Scientific, and Sigma Aldrich were used without further purification. ^{1}H and ^{13}C NMR spectra were acquired on a Bruker Avance III 500 MHz NMR spectrometer with chemical shifts reported relative to DMSO-d_6 (2.50 ppm) or CD_3OD (3.31 ppm). High resolution mass spectrometry (HRMS) was performed on a Q-Exactive (Thermo Scientific) mass spectrometer.

2-((((9H-Fluoren-9-yl)methoxy)carbonyl)amino)-3-(4-(2-(2,4-dioxopentan-3-ylidene)hydrazineyl)phenyl)propanoic acid (2). Fmoc-4-amino-L-phenylalanine (**1**, 1.01 g, 2.6 mmol) was dissolved in acetic acid (10.0 mL), cooled to 0 °C, and concentrated HCl (1.0 mL) was added. Sodium nitrite (195.2 mg, 2.8 mmol, 1.1 eq) was dissolved in water (500 µL) and this solution was added slowly dropwise to the reaction vial. The resulting solution was stirred at 0 °C for 10 minutes. Then, a solution of 2,4-pentanedione (370 µL, 3.6 mmol, 1.4 eq), sodium acetate (843.3 mg, 10.3 mmol, 4 eq), water (2 mL) and ethanol (500 µL) were added slowly dropwise. After 10 minutes of stirring, the reaction mixture was diluted with ethyl acetate (50 mL). The organic layer was collected and washed with saturated sodium bicarbonate (3 x 50 mL), then brine (50 mL). The organic layer was concentrated *in vacuo*, and the resulting residue was purified via column chromatography on silica gel (10% methanol in DCM) to yield the crystalline yellow product **2** (60%, 0.80 g). ^{1}H NMR (500 MHz, DMSO-d_6) δ 14.11 (s, 1H), 7.89 (d, 2H, *J* = 7.5), 7.66 (t, 2H, *J* = 6.7), 7.49 (d, 2H, *J* = 8.5), 7.41 (t, 2H, *J* = 6.8), 7.33 (m, 4H), 4.20 (m, 4H), 3.20 (dd, 1H, *J* = 14, 4.5), 2.90 (t, 1H, *J* = 10.6), 2.48 (s, 3H), 2.38 (s, 3H). ^{13}C NMR (125 MHz, DMSO-d_6) δ 197.0, 196.7, 173.6, 156.3, 144.2, 141.1, 140.7, 135.9, 133.6, 130.8, 128.1, 127.5, 125.7, 125.6, 120.5, 116.7, 66.1, 55.9, 47.0, 36.5, 31.6, 26.8. HRMS $(M+H)^+$ calcd for $C_{29}H_{28}O_6N_3$ 514.19726, found 514.19790.

***p*-Arylazopyrazole-phenylalanine (AAPF).** The diketone **2** (780.0 mg, 1.5 mmol) was dissolved in ethanol (20 mL) and methylhydrazine (2.4 mL, 45.6 mmol, 30 eq) was added. The solution was stirred under reflux conditions for 3 hours, then concentrated *in vacuo*. The resulting residue was recrystallized in water to yield the final product as a crystalline yellow solid (68%, 542.9 mg). ^{1}H NMR (500 MHz, DMSO-d_6) δ 7.87 (d, 2H, *J* = 8.5 Hz), 7.55 (d, 2H, *J* = 8.5 Hz), 4.32 (t, 1H, *J* = 8.5 Hz), 4.19 (s, 3H), 3.38 (m, 1H), 2.96 (dd, 1H, *J* = 14.5, 8.5 Hz), 2.81 (s, 3H), 2.63 (s, 3H). ^{13}C NMR (125 MHz, DMSO-d_6) δ 147.0, 145.6, 140.8, 129.4, 127.8, 127.4, 125.9, 121.9, 120.6, 71.3, 66.6, 56.8, 50.8, 45.4. HRMS $(M+H)^+$ calcd for $C_{15}H_{20}O_2N_5$ 302.16115, found 302.16116.

***p*-Arylazopyrazole-phenylalanine HCl salt (AAPF-HCl).** Limited solubility of **AAPF** in acetonitrile was observed, so the HCl salt was prepared by suspending the yellow solid (50 mg) in 2 mL of 4 N HCl in dioxane and stirring the solution at room temperature for 1 hour. The resultant mixture was concentrated *in vacuo* to yield the final product as the HCl salt. ^{1}H NMR (500 MHz, CD_3OD) δ 7.84 (d, 2 H, *J* = 8.5 Hz), 7.48 (d, 2 H, *J* = 8.5 Hz), 4.31 (t, 1 H, *J* = 8.5 Hz), 4.00 (s, 3 H), 3.38 (m, 1 H) 3.08 (dd, 1 H, *J* = 14.5, 8.5 Hz), 2.72 (s, 3 H), 2.65 (s, 3 H); ^{13}C NMR (125 MHz, CD_3OD) δ 171.2, 152.3, 141.7, 139.6, 137.7, 134.5, 129.7, 121.8, 56.1, 36.8, 34.7, 12.4, 8.3.

Photochemical Analysis

General. For use in biological and photochemical assays, a stock solution of **AAPF**-HCl was generated at 100 mM in DMSO and stored at –20 °C.

Photochemical analysis. A solution (250 μM) of **AAPF**-HCl in PBS was prepared by diluting 25 μL of a 10 mM stock solution in DMSO into 975 μL of PBS. The glass vial containing the working **AAPF** solution was irradiated for 10 minutes using a UV transilluminator (VWR Dual UV Transilluminator, 25 mW/cm^2, as measured with a ThorLabs power and energy meter console (PM200) with sensor (S170C)) set to 365 nm, then samples were removed for absorbance and HPLC analyses. For absorbance spectra, 100 μL of the irradiated solution was added to 96-well plate (black, clear bottom), and the absorbance was measured on a Tecan M1000Pro plate reader. For HPLC analysis, 50 μL was removed from the irradiated solution and injected on a Shimadzu HPLC with a 4.6 mm analytical column (Agilent, Zorbax SB-C18, 3.5 μm, 4.6 x 100 mm) using a gradient of acetonitrile in water (5 – 95%, 30 minutes, flow rate = 1.0 mL/min).

The vial was then irradiated with a 530 nm LED (Mouser Electronics, LUMILEDS LXML- PM01-0100, output = 130 mW/cm^2, as measured with a ThorLabs power and energy meter console (PM200) with sensor (S170C)) for 10 minutes, and again a sample was removed for both absorbance and HPLC analyses. For each condition, 20 μL was injected and absorbance was detected at the isobestic point, 280 nm. The raw absorbance and HPLC data were extracted and analyzed using Prism 8. To determine the photostationary states via HPLC, the peak area at 6.0 minutes (*cis*-isomer) and peak area at 7.9 minutes (*trans*-isomer) were extracted. The "% *cis*-isomer" was determined using the following calculation: (peak area at 6.0 minutes)/(summation of peak areas of 6.0 and 7.9 minutes)*100.

Thermal stability. To qualitatively analyze the stability of *cis*-**AAPF** in aqueous media, a 250 μM solution of **AAPF**-HCl was made by diluting 25 μL of 10 mM of AAPF in DMSO into 975 μL of either PBS, LB broth, or FluoroBrite media. An absorbance scan of the non-irradiated sample (dark) was measured relative to the DMSO blank (made with the same dilution described above) in a black, clear bottom 96-well plate using a Tecan M1000Pro plate reader. The **AAPF**-HCl solution was then isomerized to the *cis*-isomer by irradiating the sample at 365 nm for 10 minutes on a UV transilluminator. Immediately after irradiation, the sample vial was covered with foil and kept in the dark at 37 °C throughout the experiment. For each time point, 100 μL of the irradiated solution was transferred to the 96-well plate and analyzed relative to the DMSO blank.

To qualitatively determine the stability of *cis*-**AAPF** in the presence of glutathione, a 100 mM stock of glutathione was made by dissolving 307 mg of reduced glutathione in 10 mL of water. A 10 mM working solution of glutathione in either LB broth or FluoroBrite media was made by diluting 300 μL of the 100 mM glutathione into 2700 μL of the respective media. The glutathione supplemented media was used as described above to prepare a 250 μM working solution of *cis*-**AAPF** and

stability was monitored over 24 h. To prevent evaporation of the solutions during this time, plates were sealed using clear tape.

To quantify the $t_{1/2}$ of *cis*-**AAPF**, a 250 µM working solution of **AAPF**-HCl was made by diluting 25 µL of a 10 mM **AAPF**-HCl stock solution (in DMSO) into 975 µL of acetonitrile or PBS, in a glass ½ dram vial. The entire working solution was irradiated at 365 nm on a UV transilluminator for 10 minutes. Immediately after irradiating, the vial was wrapped in aluminum foil and kept dark for the remainder of the experiment. The samples were stored either in a 37 °C incubator. For the 0-hour timepoint, 50 µL of the irradiated sample was transferred to a mass spec vial, 20 µL of which was injected and analyzed by HPLC (5 – 95% acetonitrile in water, 30 min, 280 nm detection, flow rate = 1.0 mL/min). The process was repeated until the remaining percentage of *cis*-**AAPF**, calculated as described above, was below 50%.

Biological and Computational Protocols

General. Top10 cells were prepared in house. HEK293T cells were obtained from ATCC and monitored for mycoplasma contamination by testing every 3 months.

Synthetase screening in *E. coli*. A glycerol stock panel (stored at –80 °C in a 96-well deep well plate, **Table S1**) containing a variety of *Mb*PylRS mutants doubly transformed with a pBAD-sfGFP-Y151TAG reporter plasmid was utilized to screen synthetases for **AAPF** incorporation. A pipette tip was gently stabbed into the frozen stock and used to inoculate 1 mL of LB broth containing kanamycin (50 µg/mL) and tetracycline (25 µg/mL). The cultures were grown overnight at 37 °C with 250 rpm shaking to reach saturation. The next day, 2 mL expression cultures for each synthetase mutant were prepared by diluting (1:100) the saturated starter culture into LB broth containing both kanamycin and tetracycline. The expression cultures were grown at 37 °C with 250 rpm shaking until the OD_{600} reached 0.5. Next, 200 µL of the expression culture was transferred to a 96-well deep well plate (two wells per synthetase, for treatment with either DMSO or **AAPF**). To the appropriate wells, either 2 µL of DMSO or 2 µL of **AAPF**-HCl (100 mM in DMSO) was added for a final 1 mM concentration, with 1% DMSO. All wells were induced by the addition of 10 µL of 2% arabinose (final arabinose concentration of 0.1%). The deep well plate was covered with aluminum foil, and the plate was secured to the platform of a shaker and shaken overnight at 250 rpm at 37 °C. The following day, 100 µL of each culture was transferred to a black, transparent bottom 96-well plate and the absorbance at 600 nm and sfGFP fluorescence (ex. 488/5, em. 510/5 nm) was measured using a Tecan M1000 Pro plate reader. The raw fluorescence values were divided by the corresponding absorbance measurement to correct for any variation in cell density and the fluorescence/absorbance was plotted for all synthetase mutants screened with each of the three conditions.

Incorporation of AAPF in *E. coli*. The same doubly transformed glycerol stock panel was utilized for generating 2 mL starter cultures for the two identified hits, PylRS 16-5 and PylRS 20 (AzoFRS2). Cultures were grown overnight at 37 °C with 250 rpm shaking to reach saturation. The next day, 20 mL cultures for both synthetase mutants were prepared by diluting the saturated starter culture 1:100 in LB broth containing kanamycin (50 µg/mL) and tetracycline (25 µg/mL). These cultures were grown at 37 °C with 250 rpm shaking until the OD_{600} reached 0.5, then 10 mL of culture was aliquoted to 50 mL conical tubes (two per synthetase mutant). The 10 mL cultures were treated with either 100 µL of DMSO or **AAPF**-HCl for 15 minutes, followed by the addition of 25 µL of 20% arabinose for a final 0.1% arabinose concentration. The cultures were incubated with 250 rpm shaking at 37 °C overnight to allow for protein expression. The next day,

cells were pelleted at 4000 g for 10 minutes and the cell pellets were lysed following a three-phase partitioning protocol,[1] purified over Ni-NTA resin, and analyzed via 10% SDS-PAGE with a 4% stacking gel on ice (60 V for 20 minutes followed by 150 V for 80 minutes). A sample of sfGFP-Y151**AAPF** from the AzoFRS expression was also analyzed via HRMS: expected 28382.178, found 28382.063 (**Figure S2**).

Live cell imaging. HEK293T cells were plated in a black, poly-D-lysine treated, 96-well plate at 50,000 cells per well and grown at 37 °C with 5% CO_2. At ~80% confluence, cells were co-transfected with pmCherry-TAG-EGFP-HA and pAzoFRS2-4xPylT at a 1:1 plasmid ratio using 200 ng DNA in 10 µL OptiMEM per well and LPEI (Polysciences, 23966) in a 5:1 ratio of LPEI: DNA (w/w). The growth media was replaced with 100 µL DMEM (+ 10% FBS, – antibiotics) containing 0.25 mM **AAPF**-HCl (or DMSO). The transfection solution was incubated at room temperature for 20 min, then added to the wells, and the plates were incubated overnight at 37 °C with 5% CO_2. The next day, cells were gently washed 3 times with pre-warmed (37 °C) FluoroBrite media (100 µL), then FluoroBrite media was added (100 µL) for imaging. Cells were imaged using a Zeiss Axio Observer Z1 microscope equipped with a 10X Plan-Apochromat objective and EGFP (ex. 470/40, em. 525/50 nm) and mCherry (ex. 550/25, em. 605/70 nm) filter cubes. Image processing and analysis was performed in FIJI. Images were processed by using the subtract background, despeckle, and smooth functions in FIJI, then pseudocolored green to represent GFP fluorescence or red to represent mCherry fluorescence.

Western blot analysis of incorporation in mammalian cells. HEK293T cells were plated in a clear 6-well plate at 200,000 cells per well and grown at 37 °C with 5% CO_2. At ~80% confluence, cells were co-transfected with pmCherry-TAG-EGFP-HA and pAzoFRS2-4xPylT at a 1:1 plasmid ratio using 2000 ng DNA in 100 µL OptiMEM per well, and LPEI in a 5:1 ratio of LPEI: DNA (w/w). The growth media was replaced with 1 mL DMEM (+ 10% FBS, – antibiotics) containing 0.25 mM **AAPF**-HCl (or DMSO). The transfection solution was incubated at room temperature for 20 min, then added to the wells, and the plates were incubated overnight at 37 °C with 5% CO_2. The next day, cells were gently washed 3 times with 500 µL of pre-warmed (37 °C) DMEM (– phenol red, Fisher, SH3028401), then lifted with TrypLE (500 µL, Fisher, 12604021) and transferred to 1.7 mL tubes on ice. The cells were pelleted at 10,000 g for 2 min at 4 °C, then the supernatant was discarded. Cells were resuspended in 100 µL of ice-cold RIPA lysis buffer (150 mM NaCl, 1.0% NP-40, 0.5% sodium deoxycholate, 0.1% SDS, 50 mM Tris pH 8.0) with 1X Halt protease inhibitor cocktail and placed on ice on an orbital shaker for 15 minutes. Cellular debris was pelleted at 15,000 g for 7.5 min at 4 °C. Next, 72 µL of each supernatant was transferred to a 0.2 mL tube. Samples were mixed with 30 µL of 4X Laemmli sample buffer (200 mM Tris-HCl pH 6.8, 40% glycerol, 0.08% bromophenol blue, 4% β-mercaptoethanol) and boiled at 95 °C for 5 min. A portion of each sample (15 µL) was resolved on a 10% SDS- PAGE gel as described above, then transferred to a 0.45 µM PVDF membrane at 80 V for 105 minutes in Towbin buffer (25 mM Tris-HCl, 192 mM glycine, 20% methanol). Membranes were blocked for 2 hours with 5% milk in TBST (0.1% Tween-20 in 1X tris-buffered saline). One membrane was cut in half horizontally at the 55 kDa marker. The top half of the membrane was probed with anti-HA (Cell Signaling Technology, 3724S) and the bottom half was probed with anti-GAPDH (ProteinTech, 10494-1-AP). Anti-GAPDH was diluted 1:5000, while anti-HA was diluted 1:1000 each in 5% milk in TBST. The membranes were incubated with the appropriate primary antibodies overnight with rocking at 4 °C. The following day, membranes were washed three times with TBST. A secondary antibody solution (goat anti-rabbit HRP, ProteinTech, SA00001-2) was prepared using 1:10000 dilution in TBST. The secondary antibody solution was applied, and membranes were incubated at room temperature for 1 hour with rocking. Membranes were again washed three times with fresh TBST, then developed by incubating with SuperSignal West Pico PLUS Chemiluminescent Substrate for

5 minutes. Blots were imaged on a BioRad ChemiDoc system using the Chemi Hi Sensitivity setting. Images were processed using BioRad Image Lab software.

Western blot for control of translation – single irradiation. *E. coli* was doubly transformed with pBAD-sfGFP-Y151TAG and pBK-AzoFRS. Cultures were grown overnight at 37 °C with 250 rpm shaking to reach saturation. The next day, a 10 mL culture was prepared by diluting the saturated starter culture 1:100 in LB broth containing kanamycin (50 µg/mL) and tetracycline (25 µg/mL). These cultures were grown at 37 °C with 250 rpm shaking until the OD_{600} reached 0.5, then 2 mL of culture was aliquoted into each of four 15 mL conical tubes (one per treatment condition). The 2 mL cultures were treated with either 20 µL of DMSO or **AAPF**-HCl (pre-irradiated with either 365 nm or 530 nm light for 5 min) for 15 minutes, followed by the addition of 20 µL of 20% arabinose for a final 0.2% arabinose concentration. At this time, the "*cis*-**AAPF** (530 nm)" sample was removed, pipetted into a glass 6-dram vial, and irradiated with 530 nm light (5 min), then returned to the incubated shaker. The cultures were incubated with 250 rpm shaking at 37 °C overnight to allow for protein expression. After this time, 72 µL of each culture was removed and added to 30 µL 4X Laemmli sample buffer in 0.2 mL tubes, then each sample was boiled at 95 °C for 5 min. Western blot analysis was performed as described above with the exception that the entire membrane was probed with anti-His (ProteinTech, 100010AP, 1:2000 dilution).

Western blot for control of translation – pulsed irradiations. This experiment was performed as above, with the exception that the "*cis*-**AAPF** (365 nm)" sample was removed each hour for 6 hours, pipetted into a glass 6-dram vial, and irradiated with 365 nm light (5 min), then returned to the incubated shaker. After 6 hours of induction with regular irradiations, all samples were removed and analyzed by western blot as described above. Triplicate experiments were quantified using BioRad Image Lab software. Band intensity was measured for each condition, averaged, and normalized to the DMSO-treated control. Statistical analysis was performed in Prism 8.

Computational Modeling. Computational modeling was performed with PyRosetta, a Python-based interface to Rosetta. Models were scored with the ref2015 score function, with added weights for constraints to favor models with geometries that correspond to active pockets. Chemical structures for both *cis*- and *trans*-**AAPF** were created with Avogadro, and conformers for each structure were generated based on the rotamer library for phenylalanine in combination with previously published dihedral values for the dihedral angles surrounding the azo bond.[2]

To generate starting structures, the base crystal structure of the amino acyl tRNA synthetase was generated using AlphaFold2 from the corresponding FASTA sequence and PDB 4ZIB given as a template, using the default settings. The structure underwent FastRelax for initial minimization. Docking of the ATP, magnesium ions, a water molecule, and the unnatural amino acid was based on the positioning of the substrates in PDB 4ZIB. The positioning in 4ZIB was also used to generate constraints.

Models were generated via sampling of rotamers of residues and conformers of substrates to find compatible structures of the enzyme and substrates. The structures underwent FastRelax on the active site to find sets of rotamers with optimized favorability. Conformers for each substrate were sampled from the specified rotamer library and rotamers of amino acids were sampled from the databases within Rosetta. Constraints were applied to ensure the sampled conformers still adhered to the necessary geometry for an active pocket. Sampling was repeated 10 times for each initial structure. The iterations with the lowest energies were kept for comparisons.

Supporting Tables and Figures

Table S1. Relevant mutations of *Mb*PylRS in the synthetase panel and original sources.

RS	267	270	271	274	311	313	315	349	Ref
**WT**	**A**	**L**	**Y**	**L**	**N**	**C**	**M**	**Y**	**-**
**16**					A	A			[3]
**16-1**			A		A	A			
**16-2**				V	A	A			
**16-3**					A	A		F	
**16-4**			A		A	A		F	
**16-5**			M	A	A	A		F	[4]
**16-7**	T				A	A			
**16-8**	S				A	A			
**17**			M	A					
**18-1**			M	A	G	G			
**18-2**		F	M	A	G	G		F	
**19-1**					G	G		F	[5]
**20**		F		M	G	G		F	[4]

Table S2. Energies of the entire structure and the pocket in each model in REU (Rosetta Energy Units).

AzoFRS with AAPF	Total Energy (REU)	Pocket Energy (REU)
trans-**AAPF**	– 847.89	– 128.37
cis-**AAPF**	– 836.87	– 118.62

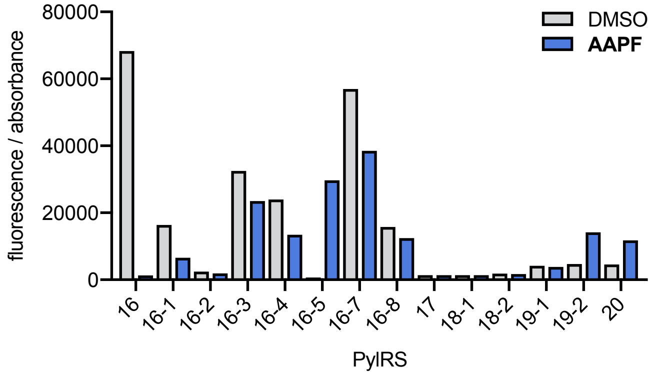

Figure S1. Select synthetases were screened for **AAPF** incorporation into the sfGFP-Y151TAG reporter and fluorescence was normalized to absorbance at 600 nm.

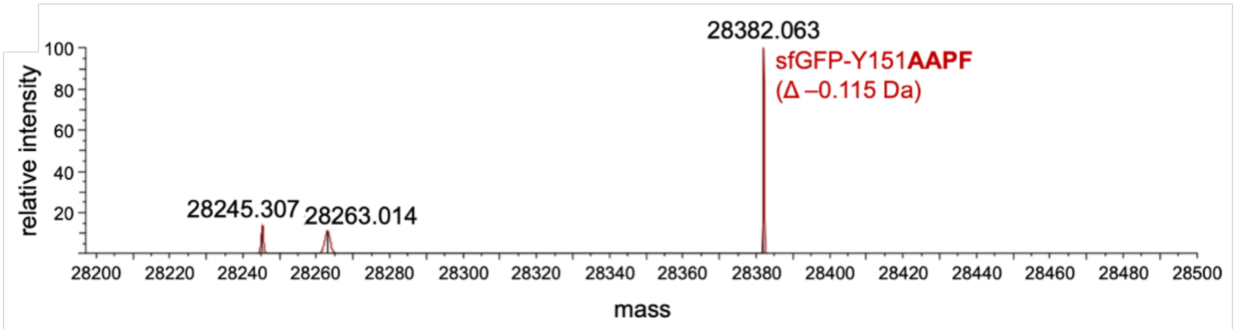

Figure S2. ESI-MS analysis of the sample shows successful incorporation with AzoFRS, with two lower abundance contaminants (28263 is likely the corresponding aniline, and 28245 represents minor background incorporation of phenylalanine).

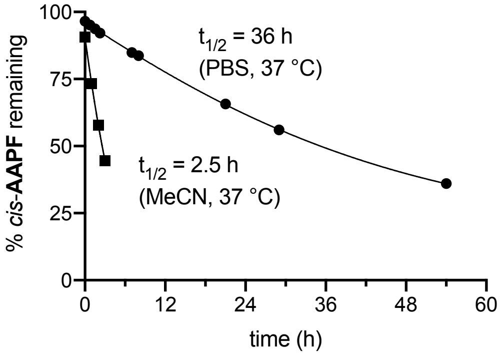

Figure S3. Half-life of *cis*-**AAPF** as determined by HPLC quantification of *cis*- and *trans*-isomers of AAPF in either acetonitrile or PBS, both stored at 37 °C.

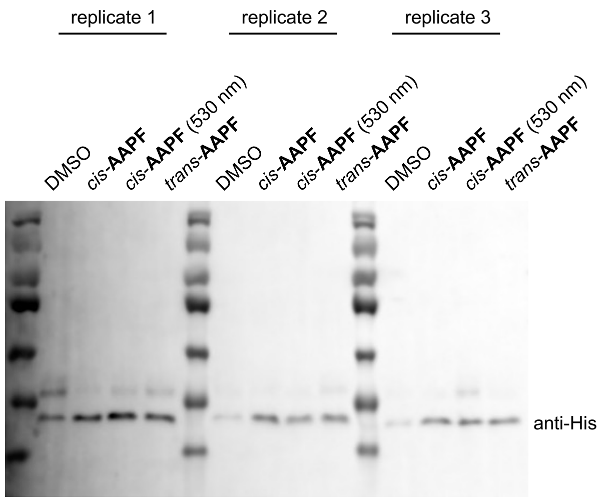

Figure S4. Triplicate western blot analysis of sfGFP-Y151**AAPF** expressed in *E. coli* cells treated with *cis*- or *trans*-**AAPF** with an overnight incubation.

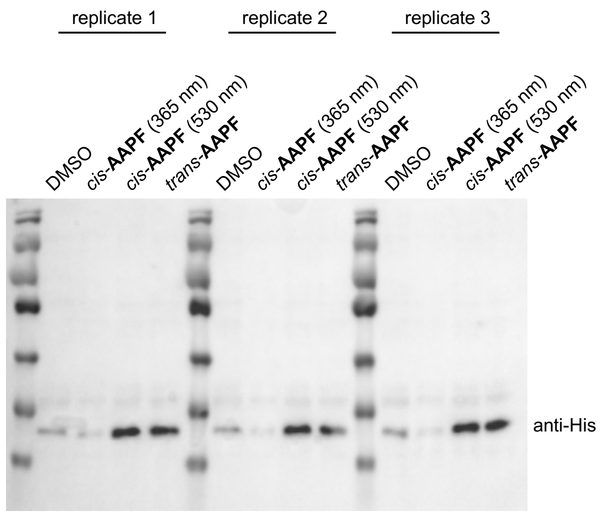

Figure S5. Triplicate western blot data used in the quantification of Figure 5C.

References

(1) Jain, S.; Singh, R.; Gupta, M. N. Purification of recombinant green fluorescent protein by three-phase partitioning. *Journal of Chromatography A* **2004**, *1035* (1), 83-86.

(2) Qianzhu, H.; Welegedara, A. P.; Williamson, H.; McGrath, A. E.; Mahawaththa, M. C.; Dixon, N. E.; Otting, G.; Huber, T. Genetic Encoding of para-Pentafluorosulfanyl Phenylalanine: A Highly Hydrophobic and Strongly Electronegative Group for Stable Protein Interactions. *Journal of the American Chemical Society* **2020**, *142* (41), 17277-17281.

(3) Lee, Y.-J.; Schmidt, M. J.; Tharp, J. M.; Weber, A.; Koenig, A. L.; Zheng, H.; Gao, J.; Waters, M. L.; Summerer, D.; Liu, W. R. Genetically encoded fluorophenylalanines enable insights into the recognition of lysine trimethylation by an epigenetic reader. *Chemical Communications* **2016**, *52* (85), 12606-12609.

(4) Luo, J.; Samanta, S.; Convertino, M.; Dokholyan, N. V.; Deiters, A. Reversible and Tunable Photoswitching of Protein Function through Genetic Encoding of Azobenzene Amino Acids in Mammalian Cells. *ChemBioChem* **2018**, *19* (20), 2178-2185.

(5) Wang, J.; Zheng, S.; Liu, Y.; Zhang, Z.; Lin, Z.; Li, J.; Zhang, G.; Wang, X.; Li, J.; Chen, P. R. Palladium-Triggered Chemical Rescue of Intracellular Proteins via Genetically Encoded Allene-Caged Tyrosine. *Journal of the American Chemical Society* **2016**, *138* (46), 15118-15121.
